# IL-22 Mediates Goblet Cell Hyperplasia and Worm Expulsion in Intestinal Helminth Infection

**DOI:** 10.1371/journal.ppat.1003698

**Published:** 2013-10-10

**Authors:** Jan-Eric Turner, Brigitta Stockinger, Helena Helmby

**Affiliations:** 1 Division of Molecular Immunology, MRC National Institute for Medical Research, Mill Hill, London, United Kingdom; 2 Department of Immunology and Infection, London School of Hygiene and Tropical Medicine, London, United Kingdom; National Institute of Allergy and Infectious Diseases (NIAID), National Institutes of Health (NIH), United States of America

## Abstract

Type 2 immune responses are essential in protection against intestinal helminth infections. In this study we show that IL-22, a cytokine important in defence against bacterial infections in the intestinal tract, is also a critical mediator of anti-helminth immunity. After infection with *Nippostrongylus brasiliensis*, a rodent hookworm, IL-22-deficient mice showed impaired worm expulsion despite normal levels of type 2 cytokine production. The impaired worm expulsion correlated with reduced goblet cell hyperplasia and reduced expression of goblet cell markers. We further confirmed our findings in a second nematode model, the murine whipworm *Trichuris muris*. *T.muris* infected IL-22-deficient mice had a similar phenotype to that seen in *N.brasiliensis* infection, with impaired worm expulsion and reduced goblet cell hyperplasia. *Ex vivo* and *in vitro* analysis demonstrated that IL-22 is able to directly induce the expression of several goblet cell markers, including mucins. Taken together, our findings reveal that IL-22 plays an important role in goblet cell activation, and thus, a key role in anti-helminth immunity.

## Author Summary

Intestinal helminth (worm) infections are some of the most common parasite infections in the world. Immunity to worm infection is dependent on the production of Type 2 cytokines, such as IL-4 and IL-13, and the induction of mucosal defence mechanisms including production of mucus by intestinal goblet cells. Here we show that the cytokine IL-22, which was previously known to be involved in the defence against bacterial infections in the gut, is also involved in the defence against intestinal worms. IL-22 deficient mice are unable to expel the rodent parasites *Nippostrongylus brasiliensis* and *Trichuris muris* from their intestines despite the fact that they make strong Type 2 cytokine responses. This inability to expel the worms correlates with a reduction in the number of goblet cells as well as a reduction in intestinal mucins and other goblet cell products. We also demonstrate that IL-22 is able to act directly on goblet cells to stimulate the secretion of mediators such as mucins. Taken together, our data show that IL-22 is a key mediator of anti-helminth immunity in the gut. Furthermore, our data provide additional insight into the pivotal role played by IL-22 in protection against various types of intestinal pathogens.

## Introduction

Type 2 immune responses are essential in protection against intestinal helminth infections, including the rodent hookworm *Nippostrongylus brasiliensis* (*Nb*) [1]. Type 2 immunity involves the recruitment of effector cells such as eosinophils, mast cells and production of IgE antibodies [2] and it is well established that the activation of IL-4- and IL-13-producing CD4^+^ T helper 2 cells is central in the development of a successful anti-parasite response. One of the key components in the expulsion of intestinal helminths is secretion of mucus by goblet cells [2]. Intestinal goblet cells are found interspersed within the epithelial monolayer and are differentiated from epithelial progenitor cells. Goblet cells produce a number of effector molecules including a range of mucins and antimicrobial proteins, including trefoil factors and resistin-like molecules, which enable these to play a key part in innate defense mechanisms in the gut, against both bacterial and helminth infections [2], [3].

IL-22 is a member of the IL-10 cytokine family and is produced by a wide variety of innate and adaptive immune cells including CD4^+^ T cells, most notably Th17 and Th22 cells, CD8^+^ T cells, natural killer (NK) cells, lymphoid tissue inducer (LTi) cells and other group 3 innate lymphoid cells (ILCs) [4]. The heterodimeric receptor for IL-22, consisting of the IL-22R and the IL-10R2 chain, is exclusively expressed on non-hematopoietic cells, such as intestinal epithelial cells, and its signaling is mediated via Stat3 [5], [6], [7], [8], [9]. IL-22 has been shown to directly mediate epithelial defence mechanisms through the induction of IL-6, IL-8 and various antimicrobial peptides [6], [10], [11], [12], [13]. Furthermore, a protective function of IL-22 has been demonstrated in some colitis models and a model of Concanavalin A induced liver damage [14], [15], [16], [17]. Therefore it appears that the function of IL-22 is to strengthen epithelial barriers, mediate repair and wound healing mechanisms as well as participate in epithelial defence. Interestingly, IL-22 has been shown to be upregulated within the human gastrointestinal tract following infections with the whipworm *Trichuris trichiuria* and the hookworm *Necator americanus* [18], [19], but no studies have as yet demonstrated a role for IL-22 in intestinal helminth infections and the associated type 2 response.

To clarify the role for IL-22 in the defence against intestinal helminth infection, we infected wild-type (WT) and IL-22-deficient mice with *Nippostrongylus brasiliensis*, a rodent nematode with a life cycle resembling that of human hookworm. Our data show that IL-22-deficient mice have reduced worm clearance, despite strong induction of IL-4, IL-5 and IL-13 in the mesenteric lymph nodes and mucosal tissue. Despite this intact type 2 cytokine induction IL-22-deficient mice showed a defective goblet cell response and *in vitro* and *ex vivo* analyses revealed that IL-22 can directly regulate the expression of several goblet cell markers. Taken together, our data suggest that IL-22 plays a key role in driving intestinal goblet cell responses *in vivo* and thus acts as an important mediator of intestinal worm expulsion.

## Materials and Methods

### Ethics statement

All animal work was approved following local ethical review by MRC National Institute for Medical Research, NIMR, Animal Procedures and Ethics Committee and was performed in strict accordance with the U. K Home Office Animals (Scientific Procedures) Act 1986 (approved H.O Project License 80/2506).

### Animals and infections

Six to nine week old male and female C57BL/6 and IL-22KO mice [20] were bred at the specific pathogen-free animal facility at the MRC National Institute for Medical Research (NIMR, London, UK). Age- and sex-matched experimental animals (3–8 per group) were infected with 500 infective *Nippostrongylus brasiliensis* (*Nb*) larvae by subcutaneous injection [21] or with 150 embryonated *Trichuris muris* (*Tm*) eggs by oral gavage [22].

### Cell culture and cytokine analyses

Mesenteric lymph nodes were removed and single cell preparations were resuspended in RPMI 1640 supplemented with 10% heat-inactivated FCS, 2 mM L-glutamine, 100 U/ml penicillin, 100 µg/ml streptomycin and 0.05 mM β-mercaptoethanol (Life technologies). Cells were cultured at 37°C and 5% CO2 in flat-bottomed 96-well plates at a final concentration of 5×10^6^/ml in a final volume of 0.2 ml/well. Cells were stimulated with *Nb* or *Tm* antigen (25 µg/ml), or plate-bound anti-CD3 antibody (mAb145-2C11, 10 µg/ml, ATCC) and cell-free supernatants were harvested after 48 hours and stored at −80°C. Cytokine analyses were carried out using a multiplex cytometric bead assay (Flowcytomix, eBiosciences). Explants of small intestine were washed extensively in ice cold PBS and cultured overnight in the same medium as above, with or without the addition of recombinant IL-22 (R&D systems). LS174T cells (kindly provided by Dr AC Williams, University of Bristol, UK) were cultured in DMEM supplemented with 10% heat-inactivated FCS, 2 mM L-glutamine, 100 U/ml penicillin and 100 µg/ml streptomycin.

### Lamina propria cell isolation and flow cytometry

After removal of Peyer's patches the small intestine was cut into 5 mm pieces and epithelial cells and intraepithelial lymphocytes were first removed by shaking gut pieces in PBS with 10% FCS, 1 mM pyruvate, 20 µM Hepes, 10 mM EDTA, 100 U/ml penicillin, 100 µg/ml streptomycin, 10 µg/ml Polymyxin B and 2 mM DTT for 30 min at 37 C. The remaining gut tissue was washed and digested using Collagenase D (Roche, 1 mg/ml) and DNAse1 (Sigma, 10 U/ml) for 45 minutes at 37°C, before being subjected to Percoll centrifugation (37.5%), followed by washing and resuspension of the isolated lamina propria leukocytes in medium. To identify innate lymphoid cells (ILC), isolated leukocytes were stained by using fluorochrome-coupled antibodies against CD45, Thy1.2, IL-7R (CD127) and a combination of lineage markers (Lin), including CD3, CD8, CD11b, CD11c, CD19, CD49b, TCR-β, TCR-γδ, NK1.1, GR-1 and Ter119. ILC were defined as CD45^+^Lin^−^Thy1.2^+^IL-7R^+^. For further characterization of ILC surface marker expression, antibodies against CD4 and NKp46 were used. For intracellular cytokine staining isolated leukocytes were restimulated with phorbol 12,13-dibutyrate (PdBU) and ionomycin (both at 0.5 µg/ml) in the presence of brefeldin A (1 µg/ml) for 2.5 h, fixed with formalin (3.8%), permeabilised with octylphenyl-polyethylene glycol (0.1%, Sigma), and stained with fluorochrome-coupled antibodies against IL-17A and IL-22. All samples were acquired on a LSRII flow cytometer (BD Biosciences) and analysed with the FlowJo software (Treestar Inc.).

### Histopathological analyses

Consecutive lengths of small intestine taken 10 cm distal to the pyloric sphincter were fixed in neutral-buffered formalin, histologically processed using standard methods, and 5 µm sections were stained for goblet cells (Periodic Schiff). The number of cells per 20 randomly selected villus-crypt units (VCU) was determined under light microscopy from at least two sections per animal.

### Real-time PCR

Tissues were harvested and stored in RNAlater (Qiagen) at −80 C until processing. RNA was purified using Trizol (Life technologies). Reverse transcription was performed using a Quantitect RT kit (Qiagen) and real time PCR was performed in an ABI 7500 sequence detection system (Applied Biosystem) using the SYBR Green PCR Master Mix (Qiagen). Results were normalised to the housekeeping gene hypoxanthine guanine phosphoribosyl transferase (HPRT).

### Statistical analyses

Significant differences (*P*<0.05) between experimental groups were determined using Student's t-test.

## Results and Discussion

### *N. brasiliensis* infection induces IL-22 expression in Th17 cells and innate lymphoid cells in the small intestine

**Figure 1:**
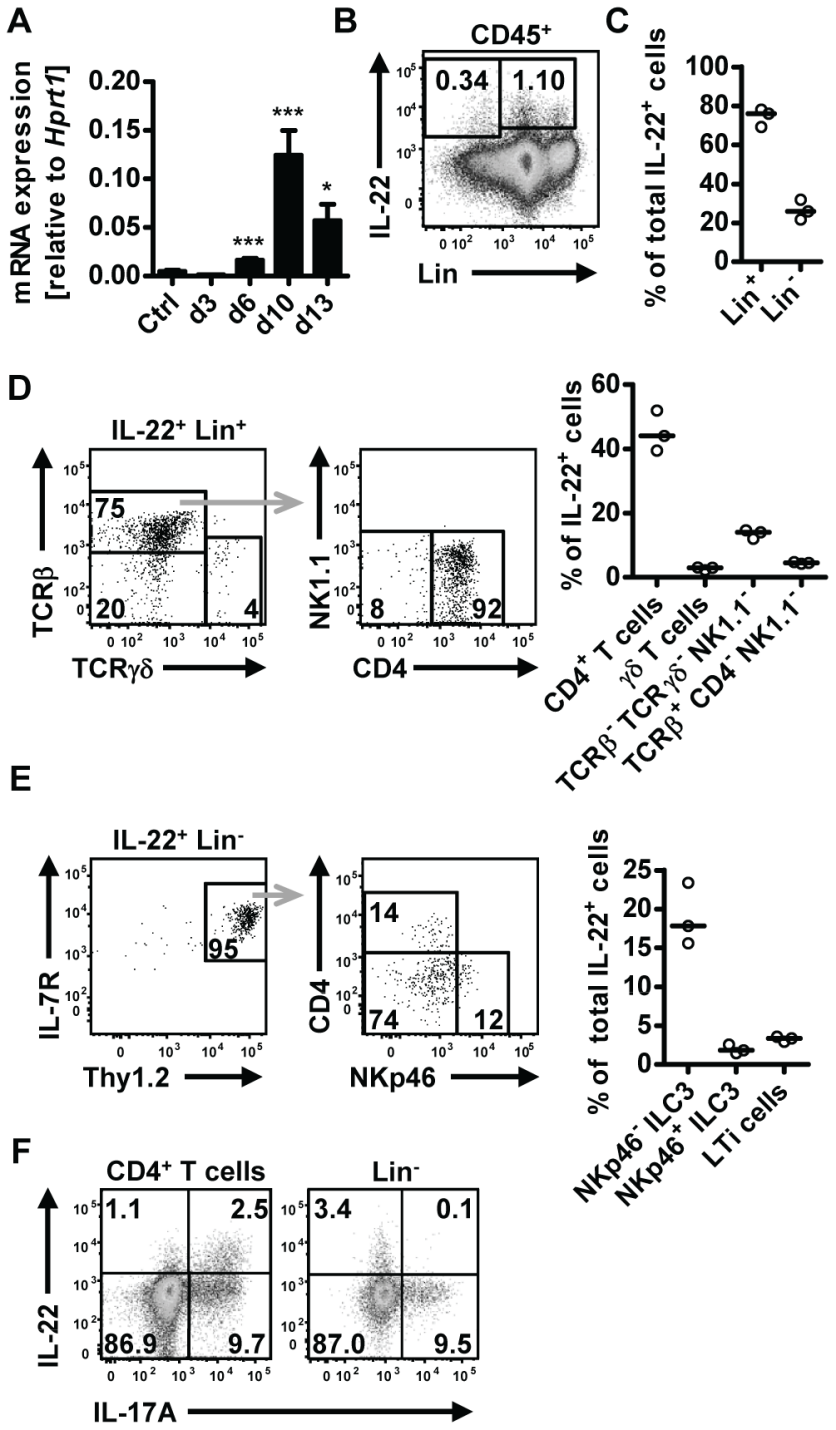
IL-22 is produced by both adaptive and innate cells in the small intestine during *N.brasiliensis* infection. (**A**) C57BL/6 mice were infected with *N. brasiliensis* and RNA was isolated from the small intestine at various time points. IL-22mRNA expression was analysed by real-time RT-PCR. Data are pooled from two independent experiments (*n* = 3–7 for each time point, and shown as mean of individual animals+SEM). (**B**) Representative flow cytometry of leukocytes isolated from the small intestinal lamina propria of infected C57BL/6 mice after restimulation with PdBU and ionomycin at day 5 post infection. (**C**) Relative contribution of Lin^+^ and Lin^−^ cells to total IL-22^+^ cells, analysed as in B. (**D, E**) Representative flow cytometry and relative contribution of the different cell subsets in the IL-22^+^Lin^+^ (**D**) and IL-22^+^Lin^−^ compartment (**E**). Relative contribution of each subset is expressed as percentage of total IL-22^+^ cells, analysed as in B. (**F**) Representative flow cytometry for IL-22 and IL-17A after restimulation with PdBU and ionomycin gated on CD4^+^ T cells (left) and Lin^−^ cells (right). Numbers in gates and quadrants represent events in percent of total cells gated. Each symbol represents one mouse. All data are representative of two to three independent experiments. *p<0.05, ***p<0.001.

Upregulation of IL-22 expression in the gastrointestinal tract has been reported in human helminth infection [18], [19]. To address the question if IL-22 may play a role in anti-helminth immunity, we utilized the mouse model of *Nippostrongylus brasiliensis* (*Nb*). We first assessed IL-22 mRNA expression in the small intestine at different time points post *Nb* infection (p.i.) (Fig. 1A). Elevated expression of *Il22* mRNA was found from day 6 p.i. and further increased at day 10 p.i. Flow cytometric analysis of lamina propria lymphocytes isolated from the small intestine of infected mice showed IL-22 cytokine staining both in the lineage-negative (Lin^−^) and lineage-positive (Lin^+^) compartments, with the majority (∼80%) of the IL-22 coming from Lin^+^ cells (Fig. 1B, C). Further characterization of the IL-22^+^Lin^+^ and IL-22^+^Lin^−^ subsets revealed that amongst the Lin^+^ cells, CD4^+^ T cells were the predominant IL-22^+^ cell type, while the IL-22^+^Lin^−^ population consisted predominantly of NKp46^−^ innate lymphoid type 3 cells (ILC3) (Fig. 1D, E). Co-staining for IL-17A demonstrated that ∼70% of the T cell-derived IL-22 was produced by Th17 cells, whereas the majority of the IL-22^+^Lin^−^ cells did not show co-production of IL-17A (Fig. 1F). Thus, our data show that IL-22 is produced by both T cell and non-T cell populations in the lamina propria during *Nb* infection. This is in agreement with a number of studies demonstrating that IL-22 can be produced by a variety of cells in the intestine, including innate cells, such as LTi, NKp46^+^ and NKp46^−^ ILC3 cells, as well as conventional CD4^+^ T cells [23], [24], [25]. In addition, Basu et al [13] recently demonstrated that early production of IL-22 during bacterial infection is mainly derived from innate sources, shifting to CD4-derived during later stages of infection, and that both sources play a vital role in protection at different stages against enteric infection, thus demonstrating the importance of both innate and adaptive sources of IL-22 in mucosal responses.

### *Il22* ^−/−^ mice show delayed worm expulsion

**Figure 2:**
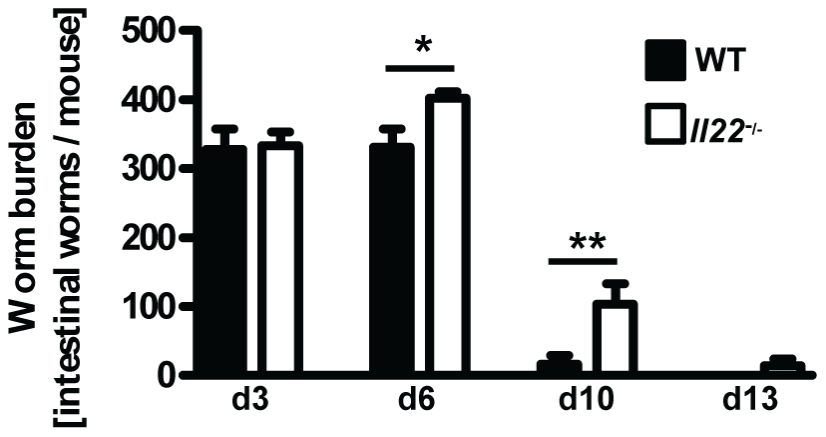
*Il22* ^−/−^ mice have reduced worm expulsion. Wild-type (black bars) and *Il22* ^−/−^ (white bars) mice were infected with *N.brasiliensis* and worm burden in the small intestine was analysed at the time points indicated. Data are pooled from three independent experiments (*n* = 5–13 for each time point, and shown as mean of individual animals +SEM). *p<0.05, **p<0.01.

Although the increased expression of IL-22 in human intestinal mucosa after helminth infections suggests a possible functional link between IL-22 and anti-helminth immunity [18], [19], the role of IL-22 in mouse models of gastrointestinal helminth infections has not been addressed. Therefore, we infected WT and *Il22* ^−/−^ mice with *Nb* and assessed the intestinal worm burdens at various time points after infection (Fig. 2). While similar numbers of parasites had reached the intestine by day 3 in WT and

*Il22* ^−/−^ mice, indicating an unperturbed lung passage of the *Nb* larvae, we observed a marked delay in worm expulsion in *Il22* ^−/−^ mice with significantly increased worm burdens at day 6 and day 10 compared to WT animals (Fig. 2), demonstrating a key role for IL-22 in the clearance of intestinal helminth infection.

### IL-22 is required for goblet cell hyperplasia after *Nb* infection

**Figure 3:**
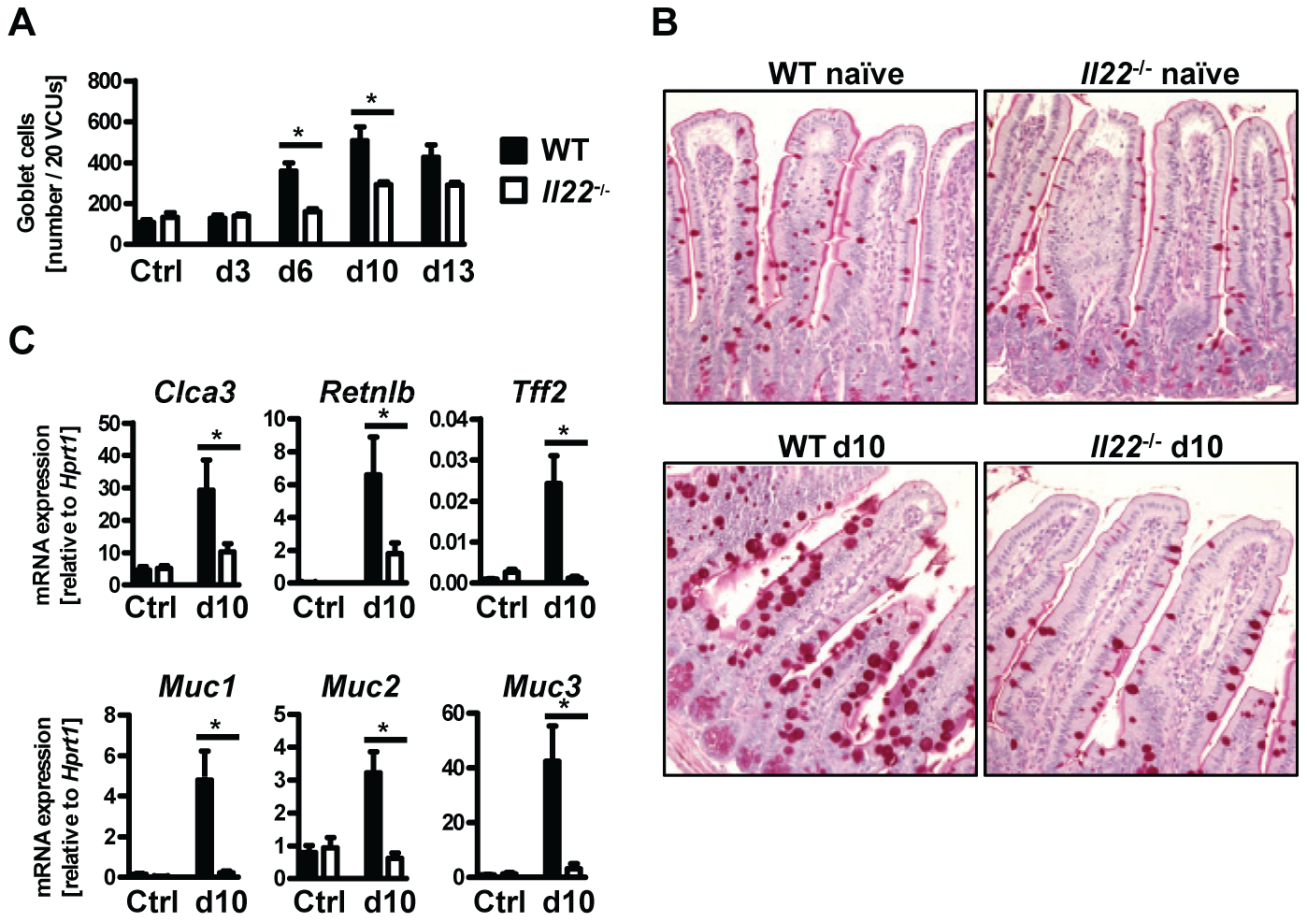
*Il22* ^−/−^ mice have defective goblet cell responses. Wild-type (black bars) and *Il22* ^−/−^ (white bars) mice were infected with *N.brasiliensis* and (**A**) lengths of jejunum collected on various days post infection, histologically processed, sectioned, stained with Periodic acid schiff and numbers of goblet cells per twenty randomly selected villus-crypt units determined by light microscopy. (**B, C**) mRNA expression measured by realtime RT-PCR in the small intestine of WT (black bars) and *Il22* ^−/−^ (white bars) mice at day 10 post infection, compared to naïve controls. All data are pooled from three independent experiments (*n* = 3–7 for each time point and shown as mean of individual animals +SEM). *p<0.05.

Goblet cell hyperplasia in the intestinal epithelium is a hallmark of intestinal helminth infections and is crucial for worm expulsion in *Nb* infection [26]. Histological analysis of the small intestine of WT and *Il22* ^−/−^ mice at various time points after *Nb* infection revealed a significant reduction of goblet cell numbers in the small intestine of *IL22^−/−^* mice as compared to their WT counterparts (Fig. 3A, B). Analysis of mRNA expression of the goblet cell markers *Clca3* (Gob5) [27], *Retnlb* (RELMβ), *Tff2* (trefoil factor 2) and mucins *Muc1*, *Muc2 and Muc3* [28], showed that their upregulation observed in *Nb*-infected WT mice was almost completely abolished in absence of IL-22 (Fig. 3C). Thus, the delayed worm expulsion observed in the absence of IL-22 is strongly correlated to reduced goblet cell hyperplasia and reduced expression of goblet cell mediators.

### Normal type 2 cytokine production in helminth-infected *Il22^−/−^* mice

**Figure 4:**
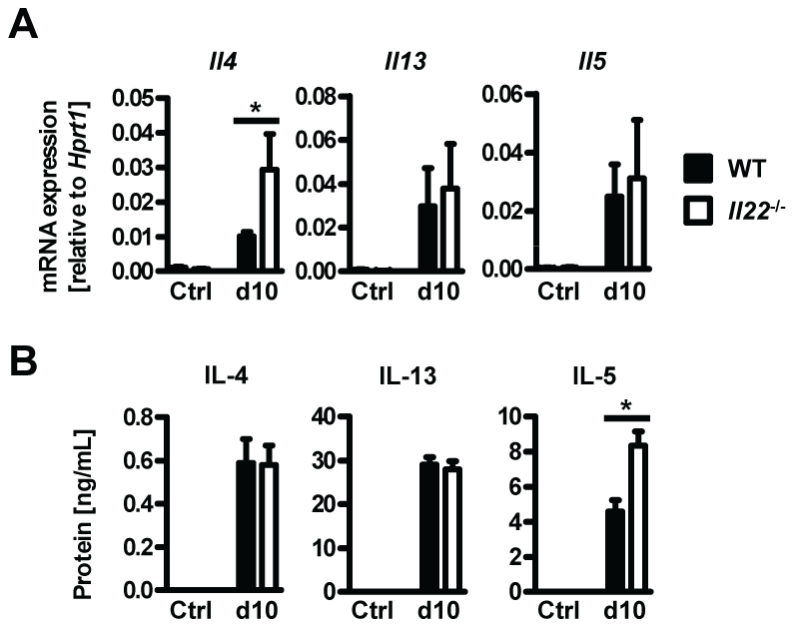
*Il22* ^−/−^ mice have normal Th2 responses. Wild-type (black bars) and *Il22* ^−/−^ (white bars) mice were infected with *N.brasiliensis* and (**A**) mRNA expression measured by realtime RT-PCR in the small intestine of WT (black bars) and *Il22* ^−/−^ (white bars) mice at day 10 post infection, compared to naïve controls. (**B**) Mesenteric lymph node cells from *Nb*-infected WT (black bars) and *Il22* ^−/−^ (white bars) mice, as well as from uninfected controls of both genotypes (Ctrl), were removed at day 10 p.i. and stimulated *in vitro* with *Nb* antigen. Supernatants were analysed by sandwich ELISA for the presence of IL-4, IL-13 and IL-5. All data are pooled from three independent experiments (*n* = 3–7 for each time point and shown as mean of individual animals +SEM). *p<0.05.

Since type 2 responses are instrumental in anti-helminth immunity and goblet cell hyperplasia, we hypothesized that impaired type 2 cytokine production may be responsible for the reduced goblet cell function and delayed worm expulsion in *Il22* ^−/−^ mice. However, neither type 2 cytokine mRNA expression in intestinal tissues, or protein levels in supernatants from *Nb* antigen-restimulated mesenteric lymph node cells at day 10 p.i. were impaired in *Il22* ^−/−^ mice (Fig. 4 A, B). In fact, the levels of IL-4 mRNA in the intestine and IL-5 protein from lymph nodes were even increased in *Il22* ^−/−^ mice, possibly as a result of overcompensation due to the increase in worm burden. Therefore, *Il22* ^−/−^ mice have impaired intestinal worm expulsion and reduced goblet cell function despite normal type 2 cytokine production.

### IL-22 acts directly on epithelial cells to induce mucin expression

**Figure 5:**
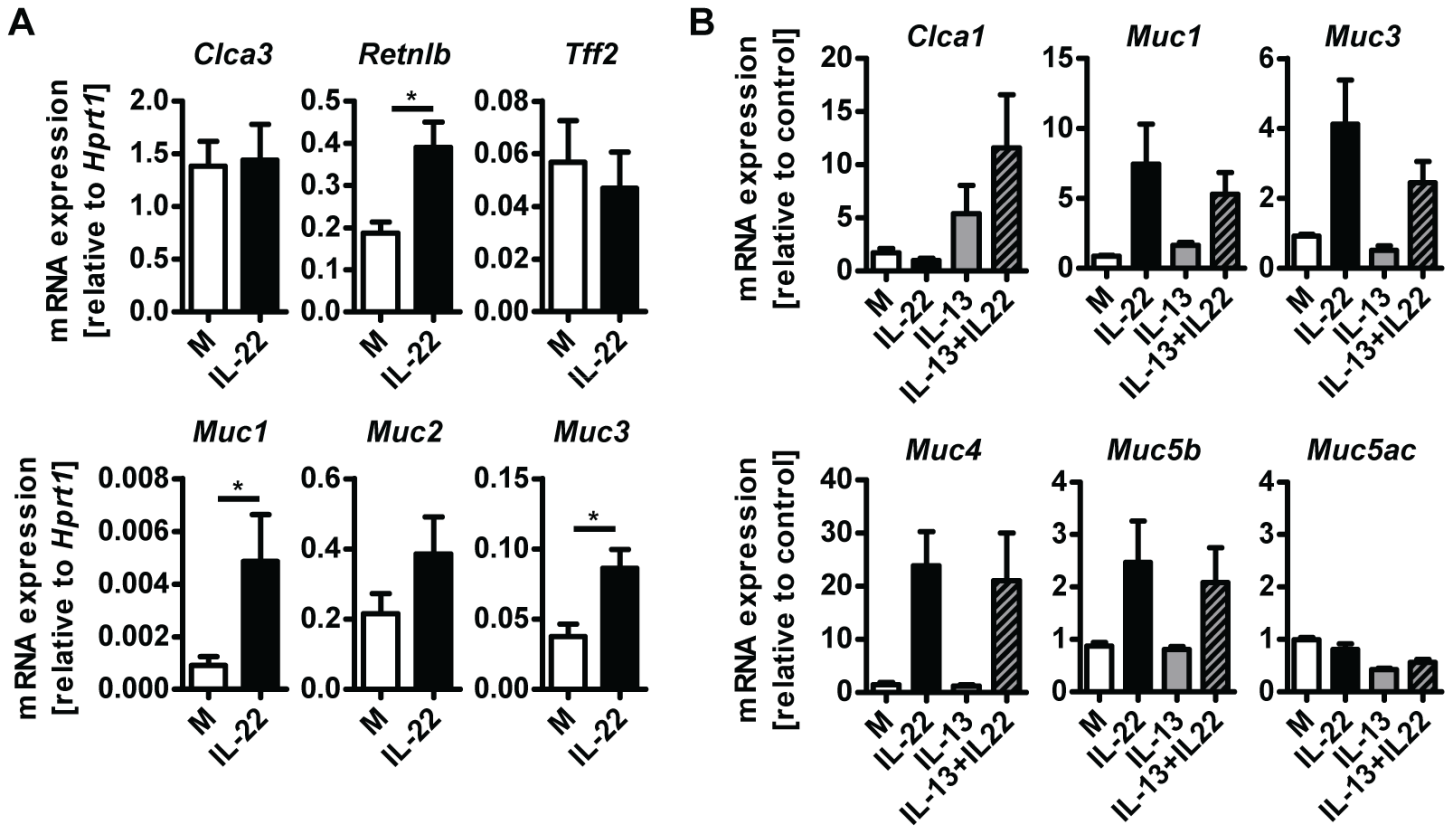
IL-22 acts directly on epithelial cells to induce mucin expression. (**A**) Segments of small intestine from naïve C57Bl/6 mice were removed, washed in ice cold PBS and incubated at 37 C overnight in complete RPMI medium with or without the addition of 10 ng/ml recombinant murine IL-22, followed by RNA isolation and analysis by real-time RT-PCR. Data are pooled from two to three independent experiments (*n* = 4 for each experiment, data shown as mean of individual animals +SEM). (B) LS174T cells were grown in complete DMEM and incubated with or without 10 ng/ml recombinant human IL-22, 10 ng/ml recombinant human IL-13, or both, for 24 hours, followed by RNA isolation and analysis by real-time RT-PCR. Data are pooled from three experiments, error bars represent variation between the independent experiments. *p<0.05.

A possible explanation for the reduction in goblet cell hyperplasia detected in *Il22* ^−/−^ mice is a direct effect of IL-22 on the differentiation and/or activation status of goblet cells. To address this possibility, we treated small intestinal tissue *ex vivo* from uninfected WT mice with IL-22 and analysed expression of goblet cell markers. IL-22 treatment induced expression of *Retnlb*, *Muc1* and *Muc3*, but not *Clca3, Muc2* or *Tff2* (Fig. 5A). In addition, we cultured LS174T cells, a human mucus-secreting intestinal adenocarcinoma cell line, in the presence of human IL-22, IL-13, or a combination of both cytokines, and observed that IL-22 alone induced expression of several mucins including *MUC1*, *MUC3*, *MUC4 and MUC5b*, but not *MUC5AC* (Fig. 5B). Similarly to our observations using mouse tissue (Fig. 5A) we did not observe induction of expression of *Clca1* (the human ortholog of mouse *Clca3*) by IL-22, however, IL-13 alone induce expression of *Clca1* and this induction was further amplified by IL-22 in a synergistic manner (Fig. 5B). Taken together, this data demonstrate that IL-22 alone is able to induce the expression of several goblet cell mediators whilst also working in synergy with other mucogenic cytokines such as IL-13, in the induction of other goblet cell mediators, such as *Clca1/3*. This data is in agreement with the study by Sugimoto et al, where IL-22 via STAT3 signaling, was linked to goblet cell hyperplasia and mucin expression in a model of colitis [15].

### *Il22* ^−/−^ mice have impaired worm expulsion and defective goblet cell responses to *Trichuris muris* infection

**Figure 6:**
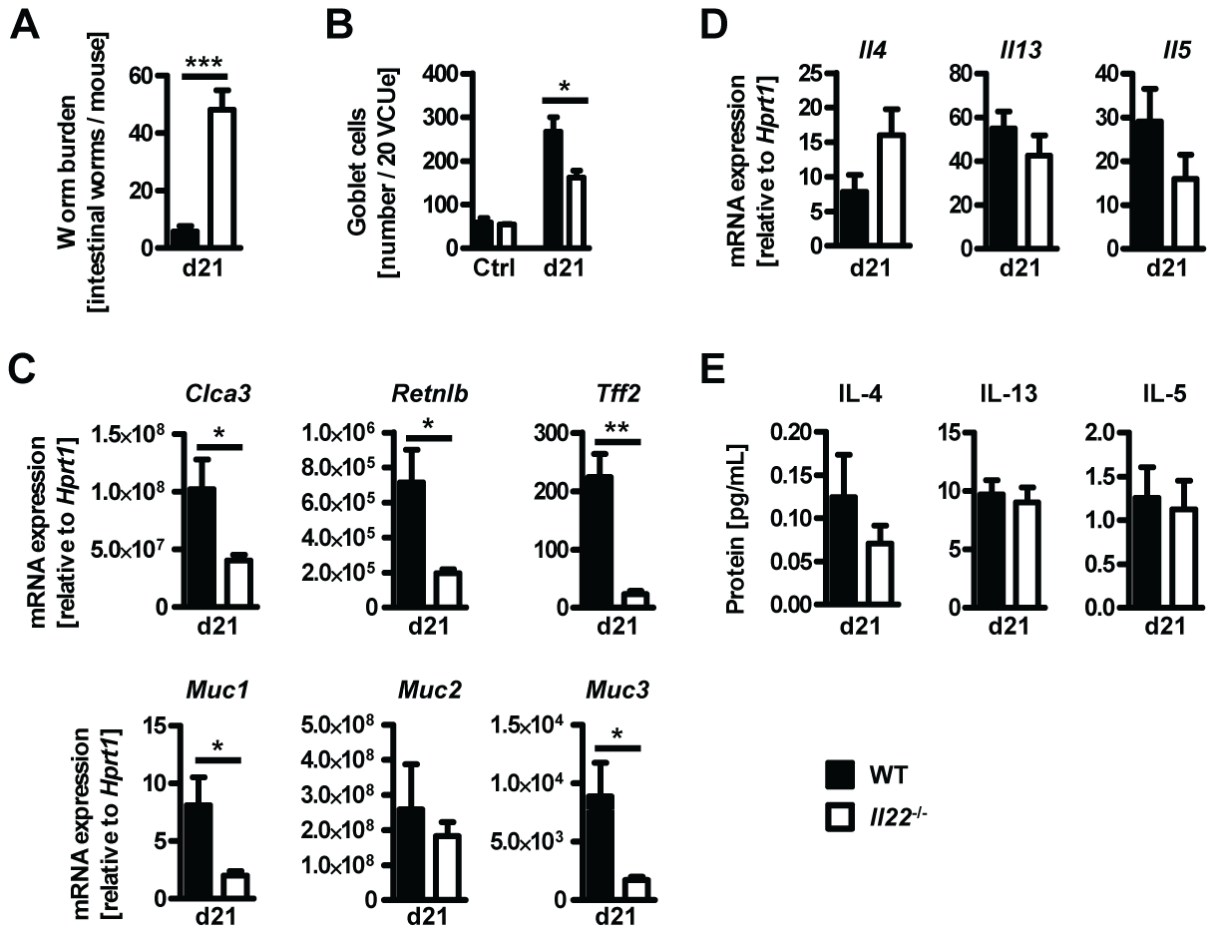
*Il22* ^−/−^ mice show reduced worm expulsion and goblet cell responses, but normal type 2 responses, during *T.muris* infection. Wild-type (black bars) and *Il22* ^−/−^ (white bars) mice were infected with *T.muris* and assessed for (**A**) worm burden, (**B**) number of goblet cells, and (**C, D**) colonic mRNA expression of goblet cell markers and cytokines by realtime RT-PCR at 21 days p.i. (**E**) Mesenteric lymph node cells were stimulated *in vitro* with *Tm* antigen and supernatants analysed by sandwich ELISA for the presence of IL-4, IL-13 and IL-5. Data from one representative experiment out of three is shown (n = 5). Data is shown as mean of individual animals +SEM. *p<0.05, **p<0.01. ***p<0.001.

In order to confirm the role for IL-22 in anti-helminth immunity we infected *Il22* ^−/−^ and WT mice with the murine whipworm *Trichuris muris*, another helminth model where goblet cells and mucus production is known to play an important role in resistance to infection [29], [30], [31]. We assessed worm burden, cytokine and goblet cell responses 21 days post infection. Similarly to *Nb* infection we found that *Tm* infected *Il22* ^−/−^ mice had higher worm burdens despite normal type 2 cytokines responses compared to WT mice (Fig. 6). Furthermore, *Tm* infected *Il22* ^−/−^ mice had reduced goblet cell hyperplasia and reduced expression of intestinal *Retnlb*, *Muc1*, *Muc3, Clca3 and Tff2*, but not *Muc2* (Fig. 6). Thus, our data demonstrate that IL-22 deficiency significantly impairs anti-helminth immunity and goblet cells function in two different murine nematode models.

Previous studies have shown that IL-22 is particularly important in regulating inflammatory responses within the intestine through the production of antimicrobial peptides, as well as enhancement and regulation of epithelial wound repair and regenerative mechanisms [6], [10], [11], [12], [13], [25]. In addition, IL-22 has also been shown to play a detrimental role in chronic mucosal inflammation and progression to colorectal cancer [32], [33] demonstrating a pivotal role for IL-22 in balancing tissue regeneration and tumour development in the intestinal environment.

Our data now suggest an important function for IL-22 in promoting other epithelial functions, particularly goblet cell-derived production of mucins and antimicrobial peptides, leading to anti-helminth immunity. A previous study by Wilson et al [34] found no role for IL-22 in the development of hepatic pathology during infection with the trematode helminth *Schistosoma mansoni* in mice, thus supporting the concept that IL-22 exerts organ and/or pathogen-specific functions. Mucus is believed to play an important part in intestinal anti-helminth mechanisms, partly through the generation of the mucus barrier, but also via other proteins in the secretions such as the antimicrobial peptide Relmβ (FIZZ2) which may impair worm movement and feeding [26], [31]. The mucins themselves are a large family of both secreted and surface bound glycoproteins, together forming the mucus layer. Quantitative and qualitative differences in mucus composition are evident during intestinal infections [35], [36] although information is still limited regarding the contribution of specific mucins to the overall physical properties of mucus and how mucus composition may change with respect to different types of infections.

Little is also known regarding the cytokine-mediated control of mucin expression, but it is clear that a number of cytokines are able to induce mucin expression, and other goblet cell products *in vitro* and *in vivo*. This includes type 2 cytokines such as IL-13 and IL-4 [37], [38], but also pro-inflammatory cytokines such as TNF and IL-1 [39], [40]. Although IL-13 and IL-4 are believed to be the key driver cytokines for the induction of goblet cell hyperplasia during helminth infections [41], [42], [43], some studies have demonstrated IL-4/13-independent goblet cell hyperplasia [44]. Furthermore, increase in intestinal Muc2 and Muc3 expression during infection with the nematode *Trichinella spiralis* has been reported in IL-4-deficient mice [45]. The data presented here, together with that of Sugimoto et al [15], now show that IL-22 is central to goblet cell hyperplasia and function in the intestine. Our analyses of *Nb* and *Tm*-infected IL-22-deficient mice demonstrate that IL-22 increases the abundance of goblet cells and is required for upregulation of mucins and other goblet cell products *in vivo*. The fact that IL-22-deficient mice mount a strong type 2 cytokine response in both draining lymph nodes and intestinal tissue provides further evidence that IL-4/13 alone are not sufficient for promoting effective goblet cell functions during intestinal helminth infection. In contrast, our *ex vivo* and *in vitro* analyses suggest that IL-22 (and not IL-13) alone might be sufficient to increase mucin production by the intestinal epithelium in certain settings. *In vivo*, however, a concerted action of the type 2 cytokines together with IL-22, and possibly other inflammatory mediators, is most likely needed for an efficient anti-helminth response. The reported findings of upregulated IL-22 expression following human infections with the whipworm *Trichuris trichiuria* and the hookworm *Necator americanus* [18], [19] indicate that IL-22 may play a similar role in human helminth infections.

In conclusion, our study demonstrates a key role for IL-22 in goblet cell function and, thus, for anti-helminth immunity in the intestine. In addition, our data provide additional insight into the pivotal role played by IL-22 in mucosal immunity in protection against various types of infectious pathogens.
